# Effectiveness of therapeutic footwear for children: A systematic review

**DOI:** 10.1186/s13047-020-00390-3

**Published:** 2020-05-13

**Authors:** Matthew Hill, Aoife Healy, Nachiappan Chockalingam

**Affiliations:** Centre for Biomechanics and Rehabilitation Technologies, Staffordshire University, Stoke on Trent, ST4 2DF UK

**Keywords:** Shoes, Orthotic Devices, Disability, Child, Adolescent, Paediatric, Mobility Limitation, Assistive Devices

## Abstract

**Background:**

It is estimated that 2% of the global childhood population is living with some form of mobility impairment. Although footwear interventions are proposed to aid ambulation, there appears to be a paucity in the understanding of the effects of therapeutic footwear. This review aims to explore the effectiveness of footwear as an intervention for mobility impairment in children.

**Methods:**

A systematic search of MEDLINE, CINAHL, PubMed, SPORTdiscus and Scopus databases were performed. Studies which focused on children with some form of mobility impairment, age of 9 months to 18 years, therapeutic footwear that allowed walking, and outcome measures that had explored biomechanical or skeletal geometry or psychosocial aspects were included in this review. Modified Downs and Black quality assessment index of randomised and non-randomised studies were used to assess the methodologies of included papers.

**Results:**

Out of 5003 articles sourced, 13 met the inclusion criteria for this review. These were grouped into two titled “corrective and “functional” based on the types of footwear used for intervention. Studies within the corrective footwear group included participants aged 11 months to 5 years with moderate congenital talipes equino varus or mobile pes planus. While using skeletal geometry as an outcome, there was a limited fair quality (level II) evidence that corrective footwear has no significant effect on the development of pes planus but may assist in the reduction of deformity in congenital talipes equino varus. The functional footwear group included participants aged 3 to 17 years, predominantly with mobile pes planus or cerebral palsy. Based on biomechanical measures as an outcome, there was a limited fair quality (level III) evidence that functional footwear alters biomechanical parameters in mobile pes planus (spatiotemporal) and cerebral palsy (spatiotemporal, kinematic). Although psychosocial outcomes were considered within two studies, the analysis was limited.

**Conclusion:**

Only a limited number of studies have explored the effects of therapeutic footwear and only in a narrow range of mobility impairments. Further high-quality research is required to improve the evidence base for the effectiveness of therapeutic footwear. This should include a wide range of mobility impairments and should focus both on physical and psychosocial outcomes.

## Background

Mobility impairment in children consists of a range of congenital or acquired conditions that may be neurological, musculoskeletal or combined in nature, representing a spectrum of moderate to severe disability [1]. Mobility impairment affects the body structure and function of a child which may lead to considerable social and health detriments [1–3]. Around 2% of the childhood population is estimated to be living with some form of mobility impairment [1–5] with a number of these conditions requiring supportive intervention to aid ambulation [6, 7].

Footwear is used as an intervention to aid ambulation in mobility-impaired children [6–10]. Footwear intended for therapeutic purposes in children consists of a broad range of designs and clinical applications including pes planus, talipes equino varus, toe walking, cerebral palsy and developmental delay [9, 11–16]. Footwear appears to be widely prescribed as an assistive device by some healthcare professionals [17], and a number of studies demonstrate that conventional footwear has significant effects on typically developing children’s gait [18, 19]. However, in contrast to other assistive aids such as ankle-foot orthoses which have seen an increase in research [20–23], there appears to be a lack of understanding on the design, effects and purpose of therapeutic footwear on children living with a mobility impairment [24–26].

A recent scoping review by the authors [24] highlighted that children’s footwear research has shown a rapid increase in the past 10 years. However, footwear intended for therapeutic purposes was represented in just a small proportion of the recent literature, with limited empirical work and no focused review exploring its effects in comparison to that of conventional children’s footwear. There was also no precise terminology to define therapeutic footwear and the specifics of its role in children living with mobility impairment. The scoping review suggested that the term children’s therapeutic footwear be used as the standard definition for footwear that is designed specifically with the purpose to support or alleviate mobility impairment in childhood. Further to this, subgroupings of therapeutic footwear were suggested dependent on the intended therapeutic role: Corrective (footwear designed to bring about the correction of congenital skeletal lower limb alignment); Accommodative (footwear designed to reduce compression and shearing stresses on children’s foot deformities through the dimensional matching of footwear to the child’s foot); and Functional (footwear designed to improve dynamic gait parameters of children with mobility impairment). In addition, the scoping review highlighted the need for a systematic assessment of the level and quality of evidence of children’s therapeutic footwear research.

The overall aim of the systematic review is to establish the effectiveness of therapeutic footwear in the treatment of mobility impairment in children.

The objectives are to:

1. systematically search the published literature to identify studies that have explored the physical or psychosocial effects of therapeutic footwear on children with mobility impairment.
2. establish the levels of evidence and quality of evidence of the available research literature concerning children’s therapeutic footwear.
3. explore the benefits and/or adverse effects of therapeutic footwear interventions.

## Methodology

The systematic review followed the PRISMA guidelines [27]. Consideration was also given to recommendations for conducting systematic reviews on paediatric participants [28, 29]. The protocol for the review was registered with PROSPERO: International prospective register of systematic reviews (CRD42018097038) [30].

### Searches

A search strategy using medical subject headings (MeSH) and free-text terms related to children and footwear were developed. Databases used in this search were MEDLINE, PubMed, CINAHL, SCOPUS, and Sportdiscus. An example of the search strategy can be found in Additional file 1. The search strategy was adapted across the databases to capture eligible articles published from database inception to February 1^st^, 2018.

### Eligibility of studies

Study designs considered for this review included randomised control trials (RCTs), non-randomised controlled trials, experimental before-after studies, prospective and retrospective comparative cohort studies, and case-control studies. Case series and case report studies were not considered for inclusion. All articles to be available in full English language text.

Participants in the included studies were infants, children and adolescents of typical walking and shod age (9 months to 18 years of age) with some form of mobility impairment (defined as a musculoskeletal or neurological condition that affects motor performance). Individuals must be able to ambulate independently or with an assistive device (e.g., arm or underarm crutches, walking frames).

Interventions included the provision of therapeutic footwear to children with a mobility impairment that facilitates and allows ambulation. Studies were included where therapeutic footwear was provided and assessed separately as an independent variable.

Therapeutic footwear that did not permit ambulation during wear (e.g., nocturnal braced footwear) were excluded. Comparators included studies that compare therapeutic footwear to barefoot, standard retail footwear, orthotic interventions and different types of therapeutic footwear.

Primary outcomes considered biomechanical and skeletal geometric measures assessing the effects of therapeutic footwear on lower limb development and function. Secondary outcomes considered measures assessing the effect of therapeutic footwear on children’s quality of life, including, physical activity, societal participation, self-esteem, and pain. Reports of adverse effects (e.g. footwear fit related pain/discomfort) in the included studies were also considered.

### Study selection

Prior to screening, all duplicates were removed using referencing software (Mendeley, Elsevier BV) and supplemented by a manual check by one reviewer (MH). Screening followed on from the previous scoping review [24], where one reviewer (MH) had independently identified studies that considered children’s footwear from a therapeutic perspective amongst the total records sourced. These abstracts were then screened by two reviewers (MH, AH) against the eligibility criteria of the systematic review with any uncertainty resolved through discussion with the third reviewer (NC). Full texts were located for all studies that appeared to meet the inclusion criteria and those studies where there was uncertainty regarding eligibility.

Two reviewers (MH and AH) independently screened the full-text articles to assess whether these met the eligibility criteria. Any disagreements regarding study eligibility between the reviewers were resolved through mediation with a third reviewer (NC).

### Data extraction

A data extraction form was developed, and information relevant to the review question was extracted from the included studies [31]. These included author names, date of publication, study design, participant characteristics (number of participants, age, sex, height, mass), description of intervention and comparison, experimental methodology, duration of follow-up, primary and secondary outcomes, adverse events and key results. Data were extracted by one reviewer (MH). The extracted data were checked for correctness and completeness against the full-text articles by a second reviewer (AH).

### Levels of evidence and quality assessment

The levels of evidence of each included study were assessed by two reviewers (MH and AH) using the Oxford Centre for Evidence-Based Medicine level of evidence version 2 (OCEBM) [32]. The quality of the studies was assessed independently by two reviewers (MH and AH). Quality assessment was completed using the modified Downs and Black quality assessment index (QI) of randomised and non-randomised studies [33, 34], which has been used in previous systematic reviews of footwear and orthoses [18, 35]. Questions that were not applicable to the study under assessment were not applied (i.e. non-longitudinal studies, studies with only one testing group). Scores were therefore adjusted to an overall percentage to mitigate for the differing total scores. In line with Trac et al. (2016) [34] the percentage scores were grouped into the following four QI levels: excellent (92 to 100%), good (71 to 91%), fair (50 to 70%), and poor (less than 50%). Survey studies were assessed separately using the tool suggested by Burns and Kho [36]; this was carried out independently by two reviewers (MH and AH).

Outcome measures from individual trials with acceptable levels of homogeneity in participant characteristics and experimental protocols were planned to be combined through meta-analysis. Where a meta-analysis was not possible, the results from clinically comparable trials were synthesised qualitatively. Data was grouped primarily on therapeutic footwear classification established in the previous scoping review [24], with subgrouping of the included studies dependent on the type of outcomes measures (biomechanical, skeletal geometry, quality of life), and pathology/medical condition.

## Results

**Fig. 1:**
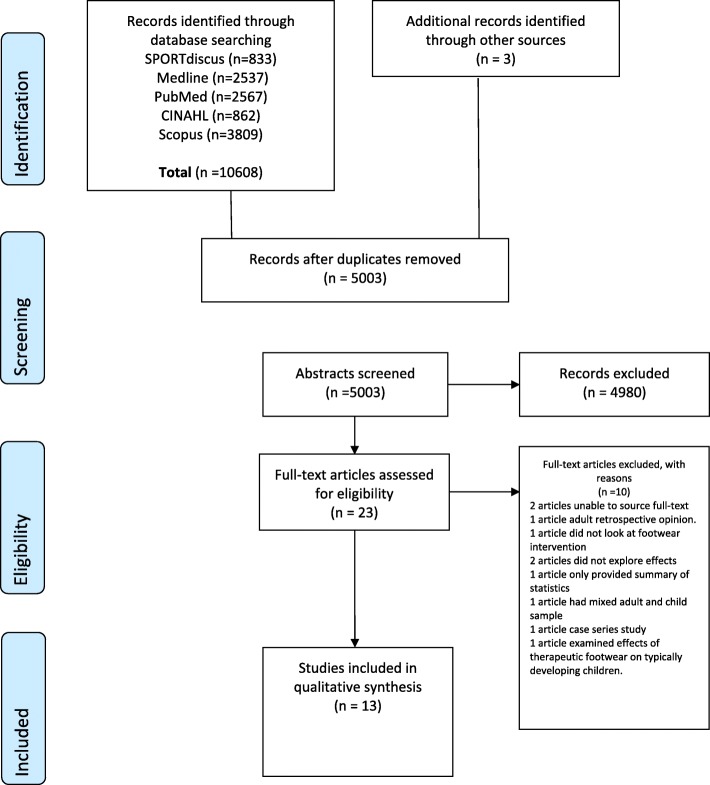
PRISMA flow diagram

Database searches yielded 5003 unique articles (Fig. 1) with 3 further papers found through screening the reference lists of related reviews sourced from the previous scoping review [24]. From these, 80 articles were identified as discussing children’s therapeutic footwear with 23 articles identified for full-text eligibility screening. Thirteen studies met the eligibility criteria for inclusion. A summary of the findings is presented in Tables 1, 2, 3, 4, 5 with supplementary results found in Additional files 2 and 3. Details of the levels of evidence and quality assessment of the included studies are provided in Additional files 4 and 5. None of the studies offered an acceptable level of homogeneity to allow the data to be combined for meta-analysis. This was due to a number of factors including, lack of sufficient detail to assure similar footwear design between studies, and incomplete description of the participants’ characteristics (Table 1 and 2). These issues precluded a combined analysis even for those studies with the same footwear grouping, clinical condition and outcomes [12, 37]; therefore, only a qualitative analysis of the included studies was possible.

**Table 1:** Details of the participants in the included studies

Study	Duration of Study	Group Intervention and Comparators	n	Condition	Sex No. (%)	Age (mean ± SD)	Mass (mean kg ± SD)	Height (mean m ± SD)	BMI (mean kg/m^**2**^ ± SD)	Outcomes
**Corrective Therapeutic Footwear**										
Chen et al. (2015) [16]	44 months	Group 1 CTF and DB	20	Group 1 to 3 CTEV (not stated if idiopathic) DiMeglio score ≤Moderate	♂12 (60)	4.9 yrs. ±1.1^a^	19.2	1.10	Not reported	Skeletal geometry (3D laser scanning)
					♀ 8 (40)		3.6	0.11		
										Biomechanical (plantar pressure)
		Group2 DB and Own footwear	15		♂9 (60)	4.7 yrs. ±0.7^a^	17.7	1.06		
					♀ 6 (40)		2.5	0.74		
		Group 3 FAS and CTF	18		♂8 (44)	4.9 yrs. ±1^a^	19.3	1.10		
					♀10 (56)		3.8	0.11		
Kanatali et al. (2016)	mean 34.6 ± 10.9 months	Group 1 CTF	21	Flexible pes planus asymptomatic,	♂33 (73)	41.6 months^c^	Not reported	Not reported	Not reported	Skeletal geometry (radiographic)
		Group 2 Own footwear	24		♀12 (27) ^b^	36 months^c^				
Wenger et al. (1989) [37]	3 years	Group 1 CTF	28^d^	Flexible pes planus	♂16 (57)	32.2 months ±17^c^	Not reported	Not reported	Not reported	Skeletal geometry (radiographic)
					♀12 (43)					
		Group 2 SLF	21^d^		13 (62)	27.2 months ±11.6^c^				
					♀8 (38)					
		Group 3 CTF with Helfet Heel Cup	27^d^		♂22 (81)	28.7 months ±13.5^c^				
					♀5 (19)					
		Group 4 SLF with UCBL	22^d^		9 (41)	28.2 months ±10.7^c^				
					♀13 (59)					
**Functional Therapeutic Footwear**										
**Functional Stability**										
Abd Elkader et al. (2013) [14]	Within 1 day	Group 1 BF, FSTF	15	Down's syndrome with flexible pes planus	♂11 (36)	3.67 yrs. ±0.72	16.46	1.01 0.069	16.01	Biomechanical (spatiotemporal)
					♀14 (47) ^b^,^e^		2.74		1.67	
		Group 2 BF, Foot Taping	15			4.06 yrs. 0.88	15.61	0.99 0.032	15.49	
							1.99		1.47	
Aboutorabi et al. (2014) [11]	Within 1 day	Group 1 BF, FSTF, SLS with FO	30	Flexible pes planus	♂18 (67)	7.87 yrs. ±1.45	31.4	123.06 10.25	20.2	Biomechanical (spatiotemporal)
					♀12 (33)		5.74		1.58	
		Group 2, BF, FSTF, SLS with FO	20	Control, typically developing	♂12 (60)	7.8 yrs. ±1.31	32.81	1.28	19.87	
					♀8 (40)		6.66	.11	1.4	
Bakker et al. (1997) [38]	16 Months	Group 1 FSTF	7	Duchenne muscular dystrophy	♂48 (100)	Age range 5 to 12^f^	Not reported	Not reported	Not reported	Secondary outcomes
		Group 2 AFO	20							
		Group 3 SF	6							
		Group 4 KAFO	5							
		Group 5 Own footwear	41							
Basta et al (1977) [39]	4 years	Group, 1, BF, FSTF, FSTF with CNP	10	Symptomatic flexible pes planus	Not reported	Age Range 6.5 to 7 years^f^	Not reported	Not reported	Not reported	Skeletal geometry (radiographic)
		Group 2, BF, FSTF, FSTF with CNP,	10							
		Group 3, BF, FSTF, FSTF with CNP, FSTF with PCNP,	10							
		Group 4, BF, S, SLF with HB, SLF with CNP, FSTFWS	6							
		Group 5 and 6 formed from groups 1 to 4, Four participants lost to follow up.								
		Group 5, BF, FSTF, FSTF with CNP Group	16							
		6, BF, CNP, with Own footwear	16							
		Group 7, BF, FSTF, FSTF with CNP,	14							
Jagadamma et al (2009) [40]	Within 1 day	One group AFO and SSF, FSTF+AFO,	5	CP	♂3 (60)	9.7 yrs. ±3.5	Not reported	Not reported	Not reported	Biomechanical (kinematic, kinetic, spatiotemporal)
					♀2 (40)	Range 5.6 to 12.6yrs.				
Knittel and Staheli (1976) [41]	Not Stated	One group, SSF, Various forefoot and Rearfoot sole wedges, Torqheel,	10	In toeing	♂4 (40)	6.25 yrs. ±2.35	Not reported	Not reported	Not reported	Biomechanical (kinematic)
					♀6 (60)	Range 3.5 to 10 yrs.				
Wesdock & Edge (2003) [42]	8 weeks	One group, SSF, SSF and AFO, FSTF+AFO	11	CP	♂4 (36)	7 yrs. ±2.7	Not reported	Not reported	Not reported	Biomechanical (spatiotemporal)
					♀7 (64)	Range 4 to 13.5 yrs.				
		Subset of Group 1 SSF, SSF and AFO, FSTF+AFO	4	CP Initial standing balance ≥15 seconds	♂3 (75)	6.5 yrs. ±2	Not reported	Not reported	Not reported	
					♀1 (25)	Range 4.6 to 9.3 yrs.				
**Functional Instability**										
Ramstrand et al (2008) [43]	8 weeks	One Group FITF 8wk training program	10	CP + other^g^	♂6 (60)	13.8 yrs. ±2.7	51.71 11.18	1.59	Not Reported	Biomechanical (balance: static, dynamic)
					♀4 (40)	Range 10 to 17 yrs.		0.11		
**Functional Lift**										
Eek et al (2017)	Within 1 day	Group 1 BF, SSF, FLTF	10	Spastic CP with LLD ≥1cm	♂6 (60)	10.9 yrs. Range 7.8 to 12.8	38.6	1.42,	Not Reported	Biomechanical (kinematic, spatiotemporal)
					♀4 (40)		Range 25.7-59.0	Range 1.24-1.52		
		Group 2 BF, SSF	10	Control typically developing	♂5 (50)	10.7yrs	35.1	1.48		
					♀5 (50)	Range 7.1 to 14	Range 18.7-49	Range 1.20-1.67		
Zabjek et al (2001) [44]	Within 1 day	One Group, BF, FLTF	46	Idiopathic scoliosis	♂9 (19.6)	12yrs. ±2	Not Reported	Not Reported	Not Reported	Skeletal Geometry (3D stereovideographic)
					♀37 (80.4)					

**♂**Male, **♀** Female *AFO* Ankle Foot Orthosis, *BF* Barefoot, *CNP* Customised Navicular Pad, *CP* Cerebral Palsy, *CTEV* Congenital Talipes Equino Varus, *CTF* Corrective Therapeutic Footwear, *DB* Dennis Brown Splinted Footwear, *FAS* Forefoot Abduction Night Shoe, *FIFT* Functional Instability Therapeutic Footwear, *FLTF* Functional Lift Therapeutic Footwear, *FSTF* Functional Stability Therapeutic Footwear, *FSTFWS* Functional Stability Therapeutic Footwear Without Steel Shank, *GMSF* Gross Motor Functioning Score, *HB* heel block, KAFO Knee Ankle Foot Orthoses, *LLD* Limb Length Difference, *PNP* Prefabricated Navicular Pad, *SF* Standing Frame, *SLF* Standard Last Footwear, *SSF* Standard Sole Footwear, *UCBL* University of California Biomechanics Laboratory custom moulded Insert, ^a^Age When tested, ^b^ Sex distribution amongst groups not reported, ^c^ Age at entry of study, ^d^Numbers at end of study, ^e^missing 17% Sex distribution not accounted for, ^f^ age range distribution amongst groups not reported, ^g^ variety of neurological and developmental conditions within group.

**Table 2:** Description of footwear interventions in included studies.

Study	Description provided of therapeutic footwear intervention (s)
**Corrective Therapeutic Footwear**	
Chen et al (2015) [16]	Orthopaedic shoe with an orthopaedic insole and hard heel cup (CTF)
Kanatli et al (2016) [12]	Custom made orthopaedic shoe, 0.5-0.9cm longitudinal arch support, 3-4mm heel wedges. (CTF)
Wenger et al (1989) [37]	Orthopaedic shoe, steel shank, Thomas heel, long medial heel counter, navicular pad (CTF)
**Functional Therapeutic Footwear**	
**Functional Stability**	
Abd Elkader et al (2013) [14]	Medical shoes same brand and model (brand/model not stated) with prefabricated arch insert (FSTF)
Aboutorabi et al (2014) [11]	Custom made, High-top shoes, wide toe box, internal heel counter, arch inlay (FSTF)
Bakker et al (1997) [38]	No details other than off the shelf orthopaedic footwear (FSTF)
Basta et al (1977) [39]	High topped, Steel Shank, firm counter (FSTF)
Jagadamma et al (2009) [40]	Custom made heel to forefoot wedged EVA sole adhesion, used alongside AFO. Wedges adjusted until shank to vertical angle reached 12°. (FSTF+AFO)
Knittel and Staheli (1976) [41]	Low cut shoe with 9 various sole modifications, medial forefoot wedge only (FSTF 1), lateral forefoot wedge only (FSTF 2), medial forefoot and medial rearfoot wedge (FSTF 3), lateral forefoot and medial rearfoot wedge (FSTF 4), lateral forefoot and lateral rearfoot wedge (FSTF 5), medial rearfoot wedge only (FSTF 6), lateral rearfoot wedge only (FSTF 7), parallel torqheel (FSTF 8), circular torqheel (FSTF 9).
Wesdock & Edge (2003) [42]	Custom made Styrofoam wedged sole adhesion, wedge = vertical distance of posterior inferior elevated heel of the unaltered shoe from the floor when subject with crouch gait stood as erect as possible. (FSTF+AFO)
**Functional Instability**	
Ramstrand et al (2008) [43]	Masai Barefoot Technologies, MBT unstable sole shoe. (FITF)
**Functional Lift**	
Eek et al (2017) [10]	12 mm EVA sole adhesion divided into two parts heel and forefoot, (FLTF)
Zabjek et al (2001) [44]	Various sole lift adhesion 5mm, 10mm,15mm, (FLTF)

**AFO** Ankle Foot Orthosis, CTF Correctional therapeutic footwear, FSTF Functional stability therapeutic footwear, FITF Functional instability therapeutic footwear, FLTF Functional lift therapeutic footwear

**Table 3:** Outcome measures **Skeletal Geometry**

Outcome	Study	Condition	Group	Baseline Mean (SD ±/-)	Final Mean (SD ±/-)	Statistical Result (Significant values given in bold)
**Corrective Therapeutic Footwear**						
3D Laser scanning						
Bean shaped ratio	Chen et al. (2015) **[**16**]**	CTEV	Group 1 CTF and DB	N/A	0.29 (0.27-0.30)^a^	One-way MANOVA: **p=0.002**
			Group 2 DB and Own footwear	N/A	0.31 (0.29-0.33) ^a^	Post hoc:
						Group 3 vs. 1 **p<0.01**
			Group 3 FAS and CTF	N/A	0.27 (0.25-0.28) ^a^	Group 3 vs. 2 **p<0.01** |
Bimalleolar angle (°)	Chen et al. (2015) **[**16**]**	CTEV	Group 1 CTF and DB	N/A	75.59 (73.98-77.21) ^a^	One-way MANOVA: **p=0.032**
			Group 2 DB and Own footwear	N/A	72.98 (69.03-6.92) ^a^	Post hoc:
						Group 2 vs. 3 **p<0.01** |
			Group 3 FAS and CTF	N/A	77.55 (75.57-79.53) ^a^	
Radiographic (Anterior-Posterior view)						
Talo calcaneal angle (°)	Kanatli et al. (2016) **[**12**]**	Mobile pes planus	Group 1 CTF	34^d^ (22-53) ^b^	23^d^ (12-37) ^b^	Wilcoxon signed rank:
						Group1 **p=0.002**; Group 2 p=**0.003**
			Group 2 Own footwear	33^d^ (20-45) ^b^	30^d^ (13-37) ^b^	Mann Whitney U:
						Group 1 vs.2 p=0.19
	Wenger et al. (1989) **[**37**]**	Mobile pes planus	Group 1 CTF	36.2 (1.2) ^c^	29.4 (0.74) ^c^	One Way ANOVA: p>0.5
			Group 2 SLF	36.3 (0.99) ^c^	31.5 (1.2) ^c^	
			Group 3 CTF with Helfet heel cup	37.1 (0.84) ^c^	30 (0.77) ^c^	
			Group 4 SLF with UCBL	36.8 (0.97) ^c^	30.1 (0.82) ^c^	
Radiographic (Lateral view)						
Calcaneal pitch (°)	Kanatli et al. (2016) **[**12**]**	Mobile pes planus	Group 1 CTF	12^d^ (2-20) ^b^	15^d^ (4-20) ^b^	Wilcoxon signed rank:
						Group 1 **p=0.002**;
						Group 2 **p=0.001**
			Group 2 Own footwear	10^d^ (1-16) ^b^	14^d^ (4-22) ^b^	Mann Whitney U:
						Group 1 vs. 2 p=0.18
Talar 1st metatarsal angle (°)	Kanatli et al. (2016) **[**12**]**	Mobile pes planus	Group 1 CTF	16^d^ (7-29) ^b^	10^d^ (0-26) ^b^	Wilcoxon signed rank:
						Group 1 **p=0.001**;
						Group 2 **p=0.001**
			Group 2 Own footwear	18.4^d^ (6-35) ^b^	9.3^d^ (0-34) ^b^	Mann Whitney U: Group 1 vs. 2 p=0.72
	Wenger et al. (1989) **[**37**]**	Mobile pes planus	Group 1 CTF	19.1 (0.75) ^c^	11.7 (0.84) ^c^	One-way ANOVA: p>0.5
			Group 2 SLF	16.7 (0.87) ^c^	11.8 (0.91) ^c^	
			Group 3 CTF with Helfet heel cup	16.8 (0.76) ^c^	11.5 (0.67) ^c^	
			Group 4 SLF with UCBL	19.7 (0.83) ^c^	11.3 (0.98) ^c^	
Talo calcaneal angle (°)	Kanatli et al. (2016) **[**12**]**	Mobile pes planus	Group 1 CTF	46^d^ (27-56) ^b^	44^d^ (32-57) ^b^	Wilcoxon signed rank:
						Group1 p=0.736;
						Group 2 p=0.113
			Group 2 Own footwear	46^d^ (34-55) ^b^	43^d^ (32-51) ^b^	Mann Whitney U:
						Group 1 vs. 2 p=0.24
Talar horizontal angle (°)	Kanatli et al. (2016) **[**12**]**	Mobile pes planus	Group 1 CTF	34^d^ (16-49) ^b^	29^d^ (19-42) ^b^	Wilcoxon signed rank:
						Group 1 **p=0.003**;
						Group 2 **p=0.001**
			Group 2 Own footwear	35^d^ (21-52) ^b^	27^d^ (21-44) ^b^	Mann Whitney U:
						Group 1 vs. 2 p=0.09
	Wenger et al. (1989) **[**37**]**	Mobile pes planus	Group 1 CTF	40.5 (0.70) ^c^	34 (0.66) ^c^	One Way ANOVA: p>0.4
			Group 2 SLF	39.8 (0.71) ^c^	34.7 (0.73) ^c^	
			Group 3 CTF with Helfet heel cup	39.5 (0.6) ^c^	34.7 (0.61) ^c^	
			Group 4 SLF with UCBL	41.8 (0.78) ^c^	34.2 (0.84) ^c^	
**Functional Stability Therapeutic Footwear**						
Radiographic (Anterior-Posterior view)						
Talocalcaneal angle (°)	Basta et al. (1977) **[**39**]**	Symptomatic mobile pes planus	Group 1 Change from BF wearing FSTF	-4.2		No Statistical test for significance performed
			Group 1 Change from FSTF wearing FSTF + CNP		-1	
			Group2 Change from BF with FSTF	-3.8		
			Group 2 Change from FSTF wearing FSTF+CNP		-1.5	
			Group 3 -6	No Data Reported	No Data Reported	
			Group 7 Change from BF wearing FSTF	-4.1		
			Group 7 Change from FSTF wearing FSTF + CNP		-1.4	
Radiographic (Lateral view)						
Calcaneal pitch (°)	Basta et al. (1977) **[**39**]**	Symptomatic mobile pes planus	Group 1 Change from BF wearing FSTF	1.8		No Statistical test for significance performed
			Group 1 Change from FSTF wearing FSTF + CNP		2.1	
			Group2 Change from BF with FSTF	1.8		
			Group 2 Change from FSTF wearing FSTF+CNP		2	
			Group 3 -6	No Data Reported	No Data Reported	
			Group 7 Change from BF wearing FSTF	2.1		
			Group 7 Change from FSTF wearing FSTF + CNP		1.55	
Longitudinal arch angle (°)			Group 1 Change from BF wearing FSTF	-2.75		No Statistical test for significance performed
			Group 1 Change from FSTF wearing FSTF + CNP		-0.9	
			Group2 Change from BF with FSTF	-2.5		
			Group 2 Change from FSTF wearing FSTF + CNP		-0.9	
			Group 3 -6	No Data Reported	No Data Reported	
			Group 7 Change from BF wearing FSTF	-2.6		
			Group 7 Change from FSTF wearing FSTF+CNP		-1.2	
Talo calcaneal angle (°)			Group 1 Change from BF wearing FSTF	0.9		No Statistical test for significance performed
			Group 1 Change from FSTF wearing FSTF + CNP		-1.35	
			Group2 Change from BF with FSTF	0.7		
			Group 2 Change from FSTF wearing FSTF + CNP		-1.25	
			Group 3 -6	No Data Reported	No Data Reported	
			Group 7 Change from BF wearing FSTF	0.8		
			Group 7 Change from FSTF wearing FSTF+CNP		-1.3	
**Functional Lift Therapeutic Footwear**						
3D stereovideographic						
Anteroposterior shift of sacral 1 (mm)	Zabjek et al. (2001) **[**44**]**	Idiopathic scoliosis	BF vs. FLTF	12 (19)	7 (5)	Paired t test: p>0.05
Anteroposterior shift thoracic 1 (mm)			BF vs. FLTF	32 (20)	7 (7)	**p<0.05**
Anteroposterior shift shoulders/pelvis (mm)			BF vs. FLTF	20 (18)	6 (5)	**p<0.05**
Diff in height left-right tibia (mm)			BF vs. FLTF	-3 (5)	11 (4)	**p<0.05**
Diff in height left-right trochanter (mm)			BF vs. FLTF	-10 (10)	15 (6)	**p<0.05**
**Kyphosis (%)**			BF vs. FLTF	7 (3)	0.6 (0.6)	p>0.05
Lateral shift sacral 1 (mm)			BF vs. FLTF	1 (10)	9 (6)	**p<0.05**
Lateral shift shoulder/pelvis (mm)			BF vs. FLTF	12 (10)	4 (3)	p>0.05
Lateral shift thoracic 1 (mm)			BF vs. FLTF	13 (15)	9 (7)	p>0.05
Lordosis (%)			BF vs. FLTF	4 (2)	0.5 (0.5)	p>0.05
Pelvic rotation (°)			BF vs. FLTF	0.4 (4)	2 (2)	p>0.05
Pelvic tilt (°)			BF vs. FLTF	3 (1)	3 (1)	**p<0.05**
Rotation shoulder/pelvis (°)			BF vs. FLTF	1 (4)	1 (1)	p>0.05
Shoulder rotation (°)			BF vs. FLTF	1 (4)	2 (2)	p>0.05
Shoulder tilt (°)			BF vs. FLTF	0.4 (2)	0.8 (0.6)	**p<0.05**
Tilt shoulder/pelvis (°)			BF vs. FLTF	-2 (2)	3 (2)	**p<0.05**
Vertical height of sacral 1 (mm)			BF vs. FLTF	897 (84)	5 (3)	**p<0.05**
Vertical height of thoracic 1 (mm)			BF vs. FLTF	1279 (117)	6 (3)	**p<0.05**
Version left iliac bone (°)			BF vs. FLTF	-11 (4)	1 (1)	**p<0.05**
Version right iliac bone (°)			BF vs. FLTF	-10 (4)	2 (1)	**p<0.05**
Diff in version right and left iliac (°)			BF vs. FLTF	-0.5 (2)	2 (1)	**p<0.05**

***BF*** Barefoot, ***CNP*** Customised Navicular Pad, ***CTEV*** Congenital Talipes Equino Varus, ***CTF*** Corrective Therapeutic Footwear, ***DB*** Denis Brown Barred Night Boot, ***FAS*** Forefoot Abduct Night Shoe, ***FLTF*** Functional Lift Therapeutic Footwear, ***N/A*** Not Applicable, ***SLF*** Standard Last Footwear, ***SSF*** Standard Sole Footwear, ***UCBL*** University of California Biomechanics Laboratory, ^a^95% Confidence Interval, ^b^Min-Max, ^c^Standard Error, ^d^Median,

**Table 4:** Outcome measures **Biomechanical**

Outcome	Study	Condition	Group	Baseline Mean (SD ±/-)	Final Mean (SD ±/-)	Statistical Result (Significant values given in bold)
**Corrective Therapeutic Footwear**						
Plantar pressure						
Average peak pressure (kPa): Lateral midfoot	Chen et al. (2015) **[**16**]**^a^	CTEV	Group 1 CTF and DB	N/A	62.21 (53.35-71.06) ^b^	One-way MANOVA: **p=0.005**
			Group2 DB and Own footwear	N/A	94.97 (66.38-123.59) ^b^	Post hoc:
						Group 1 vs. Group 2 **p<0.01**
			Group 3 FAS and CTF	N/A	60.9 (49.26-72.54) ^b^	Group 2 vs. Group 3 **p<0.01**
Maximum peak pressure (kPa): Hindfoot			Group 1 CTF and DB	N/A	148.71 (135.49-161.94) ^b^	One-way MANOVA: **p<0.001**
			Group2 DB and Own footwear	N/A	105.51 (85.73-125.29) ^b^	Post hoc:
						Group 1 vs Group 2 **p<0.01**
			Group 3 FAS and CTF	N/A	164.05 (148.22-179.90) ^b^	Group 2 vs. Group 3 **p<0.001**
Peak pressure ratio: Heel/forefoot			Group 1 CTF and DB	N/A	0.72 (0.58-0.87) ^b^	One-way MANOVA:
						**p=0.009**
			Group2 DB and Own footwear	N/A	0.44 (0.29-0.58) ^b^	Post hoc
			Group 3 FAS and CTF	N/A	0.73 (0.61-0.86) ^b^	Group 1 vs. Group 2 **p<0.01;**
						Group 2 vs. Group 3 **p<0.01**
Peak pressure ratio: Heel/lateral midfoot			Group 1 CTF and DB	N/A	1.45 (1.19-1.72) ^b^	One-way MANOVA:
						**p<0.001**
			Group2 DB and Own footwear	N/A	0.77 (0.47-1.08) ^b^	Post hoc:
			Group 3 FAS and CTF	N/A	1.98 (1.68-2.29) ^b^	Group 1 vs. Group2 **p<0.01;**
						Group 1 vs. Group 3 **p<0.01**;
						Group 2 vs. Group3 **p<0.001**
**Functional Stability Therapeutic Footwear**						
Kinematic						
Angle of gait (°)	Knittel and Staheli (1976) **[**41**]**	In toeing	SSF	- 17.3 (11.9)		ANOVA:
						**p<0.05**
			FSTF1		- 18.3 (12.4)	Post hoc
			FSTF2		- 17.7 (13.9)	FSTF1 vs. SSF **p<0.05**
			FSTF3		- 16.7 (12.7)	
			FSTF4		- 17.1 (12.5)	FSTF7 vs. SSF **p<0.05**
			FSTF5		- 16.7 (14.2)	
			FSTF6		- 17.0 (14.3)	FSTF8 vs. SSF **p<0.05**
			FSTF7		- 16.9 (12.4)	
			FSTF8		- 15.6 (14.1)	FSTF9 vs. SSF **p<0.05**
			FSTF9		- 10.7 (14.9)	
Max. knee extension (°) stance	Jagadamma et al. (2009) **[**40**]**	Cerebral palsy	AFO and SSF	- 2.6 (2.8)		Wilcoxon signed rank: **p=0.04**
			FSTF+AFO		3.7 (3.3)	
Knee flexion (°) initial contact			AFO and SSF	13.7 (8.4)		p=0.14
			FSTF+AFO		17.2 (5.1)	
Max. knee flexion (°) stance			AFO and SSF	19.7 (9.3)		p=0.06
			FSTF+AFO		25.2 (5.3)	
Shank to vertical angle (SVA) (°)			AFO and SSF	5.6 (3)		**p=0.005**
			FSTF+AFO		10.8 (1.8)	
Kinetic						
Peak knee flexion moment (N m) stance	Jagadamma et al. (2009) **[**40**]**	Cerebral palsy	AFO and SSF	0.59 (0.31)		Wilcoxon signed rank: p=0.25
			FSTF+AFO		0.7 (0.32)	
Peak Knee extension moment (N m) stance			AFO and SSF	- 0.44 (0.2)		p=0.14
			FSTF+AFO		- 0.29 (0.24)	
Spatiotemporal						
Base of support (cm)	Abd Elkader et al. (2013) **[**14**]**	Mobile pes planus	Group 1 BF	11.80 (1.06)		Paired t test:
			Group 1 FSTF		9.10 (1.31)	Group 1 **p<0.05;**
						Group 2 **p<0.05**
			Group 2 BF	12.63 (1.96)		Independent t test
			Group 2 FT		9.20 (1.17)	BF p=0.12;
						FSTF vs. FT p=0.86
Cadence (Steps/min)	Jagadamma et al. (2009) **[**40**]**	Cerebral palsy	AFO and SSF	122.5 (16.6)		Paired t test:
			FSTF+AFO		122.3 (12.4)	p=0.97
CoP displacement (mm)	Aboutorabi et al. (2014) **[**11**]**	Mobile pes planus	BF	6.55 (6.40)		Repeated measures ANOVA:
						**p=0.016**
			FSTF		5.84 (6.15)	Post hoc:
			SLS+FO		5.87 (6.40)	FSTF vs. BF **p<0.05**
Standing balance (s)	Wesdock and Edge (2003) **[**42**]**	Cerebral palsy	Group1 SSF (after 4 weeks wear of solid AFO)	11 (13)		Mixed model maximum likelihood estimate: p>0.05
		Crouch gait	Group 1 SSF + AFO (after 4 weeks wear of solid AFO)	18 (23)		
			Group 1 FSTF+AFO (after 4 weeks wear of solid AFO)	50 (68)		
			Group 1 SSF (after 4 weeks wear of FSTF+AFO)		14 (23)	
			Group 1 SSF + AFO (after 4 weeks wear of FSTF+AFO)		11 (24)	
			Group 1 FSTF+AFO (after 4 weeks wear of FSTF+AFO)		49 (70)	
Difference in standing balance (s)	Wesdock and Edge (2003) **[**42**]**	Cerebral palsy	Group 1 SSF vs. SSF+AFO (after 4 weeks wear of solid AFO)	(-6)-20 ^b^		No Statistical test for significance performed
			Group1 SSF+AFO vs. FSTF+AFO (after 4 weeks wear of solid AFO)	(-2)-66^b^		
			Group1 SSF vs. FSTF+AFO (after 4 weeks wear of solid AFO)	7 -73^b^		
			Group1 SSF vs. SSF+AFO (after 4 weeks wear of solid AFO)		(-19)-13^b^	
			Group 1 SSF+AFO vs. FSTF+AFO (after 4 weeks wear of solid AFO)		3-73 ^b^	
			Group1 SSF vs. FSTF+AFO (after 4 weeks wear of solid AFO)		0-70 ^b^	
		Cerebral palsy	SSF vs. SSF+AFO (after 4 weeks wear of solid AFO)	14 (6)		after 4 weeks wear of solid AFO
		Subset of Group1 all participants who could stand ≥15s	SSF+AFO vs. FSTF+AFO (after 4 weeks wear of solid AFO)	84 (41)		SSF vs. FSTF+AFO **p<0.05;**
			SSF vs. FSTF+AFO (after 4 weeks wear of solid AFO)	98 (47)		SSF+AFO vs. FSTF+AFO **p<0.05**;
			SSF vs. SSF+AFO (after 4 weeks wear of FSTF+AFO)		- 8 (7)	after 4 weeks wear of solid FSTF+AFO
			SSF+AFO vs. FSTF+AFO (after 4 weeks wear of FSTF+AFO)		101 (25)	SSF vs. FSTF+AFO **p<0.05;**
			SSF vs. FSTF+AFO (after 4 weeks wear of FSTF+AFO)		93 (33)	SSF+AFO vs. FSTF+AFO **p<0.05**
						(Sig based on 95% Confidence Interval of Group 1 differences in standing balance)
Step length (cm)	Abd Elkader et al. (2013) **[**14**]**	Down’s Syndrome mobile pes planus	Group 1 BF	26.53 (3.72)		Paired t test:
			Group1 FSTF		30.83 (4.28)	Group 1 **p<0.05**
						Group 2 **p<0.05**
			Group 2 BF	25.63 (4.62)		Independent t test:
			Group 2 FT		30.73 (5.51)	BF Group 1 vs. 2 p=0.62;
						FSTF vs. FT p=0.95
	Aboutorabi et al. (2014) **[**11**]**	Mobile pes planus	BF	37.99 (3.82)		Repeated measures ANOVA: p=0.478
			FSTF		38.85 (4.97)	
			SLS+FO		39.05 (4.68)	
Step symmetry (%)	Aboutorabi et al. (2014) **[**11**]**	Mobile pes planus	BF	-4.90 (4.66)		Repeated measures ANOVA: **p=0.000**
			FSTF		-2.70 (25.54)	Post hoc
			SLS+FO		16.08 (31.25)	FSTF vs. SLS+FO **p<0.05**
Step width (cm)	Aboutorabi et al. (2014) **[**11**]**	Mobile pes planus	BF	8.87 (1.61)		Repeated measures ANOVA: p=0.170
			FSTF		8.91 (1.99)	
			SLS+FO		9.41 (1.69)	
Stride length (m)	Abd Elkader et al. (2013) **[**14**]**	Down’s Syndrome mobile pes planus	Group 1 BF	0.448 (0.06)		Paired t test:
			Group 1 FSTF		0.504 (0.064)	Group 1 **p<0.05**
						Group 2 **p<0.05**
			Group 2 BF	0.455 (0.071)		Independent t test:
			Group 2 FT		0.524 (0.078)	BF Group 1 vs. 2 p=0.82;
						FSTF vs. FT p=0.44
	Jagadamma et al. (2009) **[**40**]**	Cerebral palsy	AFO and SSF	1.08 (0.19)		Paired t test: p=0.54
			FSTF+AFO		1.06 (0.20)	
Velocity (m/s)	Abd Elkader et al. (2013) **[**14**]**	Down’s Syndrome mobile pes planus	Group 1 BF	0.674 (.059)		Paired t test:
			Group 1 FSTF		0.775 (0.035)	Group 1 **p<0.05**
						Group 2 **p<0.05**
			Group 2 BF	0.672 (0.109)		Independent t test:
			Group 2 FT		0.762 (0.090)	BF Group 1 vs. 2 p=0.95;
						FSTF vs. FT p=0.61
	Aboutorabi et al. (2014) **[**11**]**	Mobile pes planus	BF	0.727 (0.136)		Repeated measures ANOVA: **p=0.000**
			FSTF		0.847 (0.156)	Post hoc:
			SLS+FO		0.779 (0.128)	FSTF vs. BF **p<0.05;**
						SLF +FO vs. BF **p<0.05**
	Jagadamma et al. (2009) **[**40**]**	Cerebral palsy	AFO and SSF	1.08 (0.1)		Paired t test: p=0.80
			FSTF+AFO		1.07 (0.14)	
**Functional Instability Therapeutic Footwear**						
Balance (Dynamic)						
Anterior posterior control (CoP)	Ramstrand et al. (2008) **[**43**]**^a^	Cerebral Palsy + mixed developmental disability	BF Medium (at 4 weeks)		45.7 (25.5-66.5) ^b^	Wilcoxon signed rank
			FITF Medium (at 4 weeks)		51.44 (33.7-69.2) ^b^	BF vs. FITF Medium at week 4 **p<0.05**
Mediolateral control (CoP)			BF Slow (baseline)	57.2 (47.0-67.2) ^b^		Friedman ANOVA:
						BF Slow **p<0.05**
			BF Medium (baseline)	66.4 (52.6-80.1) ^b^		Post hoc
						BF Slow at week 8 vs. week 4 and baseline **p<0.05**
						Wilcoxon signed rank
			BF Slow (at 4 weeks)		69.2 (59.9-78.5) ^b^	BF vs. FITF Slow at 8 weeks **p<0.05;**
			BF Medium (at 4 weeks)		75 (67.4-82.6) ^b^	BF vs. FITF Medium at 4- and 8-weeks **p<0.05**
			FITF Slow (at 4 weeks)		55.1 (36.3-73) ^b^	
			FITF Medium (at 4 weeks)		67 (54.3-79.2) ^b^	
			BF Slow (at 8 weeks)		74.89 (64.9-84.8) ^b^	
			BF Medium (at 8 weeks)		72.44 (55.1-89.9) ^b^	
			FITF Slow (at 8 weeks)		57.56 (40.3-74.8) ^b^	
			FITF Medium (at 8 weeks)		65.33 (44.5-86.2) ^b^	
Number of falls toes up condition			Subject 1,2,6,9,10	0		Chi Square:
			Subject 3	2		Between testing occasions **p<0.05**
			Subject 4	3		
			Subjects 5,8	4		
			Subject 7	10		
			Subjects 1,5, 8 -10 (at 4 weeks)		0	
			Subjects 2, 6 (at 4 weeks)		Did not participate	
			Subjects 3 ,4 (at 4 weeks)		1	
			Subject 7 (at 4 weeks)		2	
			Subjects 1,2, 4 - 10 (at 8 weeks)		0	
			Subject 3 (at 8 weeks)		1	
**Functional Lift Therapeutic Footwear**						
Kinematic						
Ankle dorsiflexion at initial contact (°)	Eek et al. (2017) **[**10**]**	Cerebral palsy	BF Long leg	-2.3^d^ (7.9) _e_		Wilcoxon signed rank:
			BF Short leg	-9.2^d^ (13.6) _e_		Comparison long to short
			FLTF Long leg		4.3^d^ (9.1) _e_	BF **p = 0.009;**
			FLTF Short leg		-2^d^ (17) _e_	FLTF **p= 0.017;**
			SSF Long leg		3.5^d^ (9.) _e_	SSF **p=0.009**
			SSF Short leg		-6.2^d^ (11.3) _e_	
Ankle dorsiflexion in stance (°)			BF Long leg	11.9^d^ (11.6) _e_		Comparison long to short
			BF Short leg	6.5^d^ (6.4) _e_		BF p = 0.22;
			FLTF Long leg		15.1^d^ (4.9) _e_	FLTF p=0.241;
			FLTF Short leg		14.4^d^ (8.6) _e_	SSF **p=0.022**
			SSF Long leg		16.5^d^ (2.8) _e_	
			SSF Short leg		11.4^d^ (10.7) _e_	
Ankle dorsiflexion in swing (°)			BF Long leg	3.7^d^ (5.8) _e_		Comparison long to short
			BF Short leg	3.2^d^ (5.5) _e_		BF **p = 0.007;**
			FLTF Long leg		6.5^d^ (10.9) _e_	FLTF **p=0.037;**
			FLTF Short leg		2.6^d^ (9.3) _e_	SSF p=0.13
			SSF Long leg		5.8^d^ (7.8) _e_	
			SSF Short leg		0.5^d^ (10.7) _e_	
Hip adduction in stance (°)			BF Long leg	8.4^d^ (6.4) _e_		Comparison long to short
			BF Short leg	7.4^d^ (4.4) _e_		BF p = 0.959;
			FLTF Long leg		6.6^d^ (2.9) _e_	FLTF p=0.646;
			FLTF Short leg		9.3^d^ (7.5) _e_	SSF p=0.646
			SSF Long leg		7.0^d^ (4.8) _e_	
			SSF Short leg		6.3^d^ (4.8) _e_	
Hip extension in stance (°)			BF Long leg	9.6^d^ (6.2) _e_		Comparison long to short
			BF Short leg	11.3^d^ (3.7) _e_		BF p = 0.114
			FLTF Long leg		12.8^d^ (8) _e_	FLTF p=0.241
			FLTF Short leg		12.3^d^ (5.70_e_	SSF p=0.203
			SSF Long leg		11.9^d^ (7.3) _e_	
			SSF Short leg		12.5^d^ (5.7) _e_	
Hip flexion at initial contact (°)			BF Long leg	36.3^d^ (9.1) _e_		Comparison long to short
			BF Short leg	29.8^d^ (5.1) _e_		BF **p = 0.005;**
			FLTF Long leg		34.9^d^ (5.4) _e_	FLTF p=0.139;
			FLTF Short leg		34.1^d^ (4.1) _e_	SSF **p=0.005**
			SSF Long leg		36.3^d^ (4.3) _e_	
			SSF Short leg		30.5^d^ (8.3) _e_	
Hip flexion in swing (°)			BF Long leg	37.3 (6.9) _e_		Comparison long to short
			BF Short leg	33.0 (5.5) _e_		BF **p = 0.009;**
			FLTF Long leg		38.7 (7.3) _e_	FLTF p=0.139;
			FLTF Short leg		36.9 (6.1) _e_	SSF **p=0.028**
			SSF Long leg		36.3 (7.5) _e_	
			SSF Short leg		33.3 (6.4) _e_	
Knee extension in stance (°)			BF Long leg	7.0^d^ (9.6) _e_		Comparison long to short
			BF Short leg	4.8^d^ (12.6) _e_		BF **p = 0.007;**
			FLTF Long leg		4.9^d^ (10.2) _e_	FLTF **p=0.028;**
			FLTF Short leg		1.9^d^ (10.9) _e_	SSF **p=0.007**
			SSF Long leg		8.8^d^ (10.6)	
			SSF Short leg		1.6^d^ (8.7) _e_	
Knee flexion at initial contact (°)			BF Long leg	13.4^d^ (6.8) _e_		Comparison long to short
			BF Short leg	11.9^d^ (7.8) _e_		BF p = 0.508;
			FLTF Long leg		7.7^d^ (7.5) _e_	FLTF p=0.114;
			FLTF Short leg		9.4^d^ (6.7) _e_	SSF p=0.386;
			SSF Long leg		7.3^d^ (11.5) _e_	
			SSF Short leg		8.10^d^ (7.5) _e_	
Knee flexion in swing (°)			BF Long leg	63.8^d^ (5.0) _e_		Comparison long to short
			BF Short leg	62.2^d^ (12.7) _e_		BF p = 0.203;
			FLTF Long leg		64.2^d^ (5.2) _e_	FLTF p=0.445;
			FLTF Short leg		60.8^d^ (13.4) _e_	SSF p=0.093
			SSF Long leg		65.6^d^ (2.7) _e_	
			SSF Short leg		62.5^d^ (15.3) _e_	
Spatiotemporal						
Cadence steps/min	Eek et al. (2017) **[**10**]**	Cerebral palsy	BF	100.6^d^ (17.8) _e_		Friedman ANOVA: p>0.05
			FLTF		98.4^d^ (25.7) _e_	
			SSF		99.3^d^ (24.9) _e_	
Stance phase %			BF Long leg	61.1^d^ (2.03) _e_		Wilcoxon signed rank:
			BF Short leg	56.8^d^ (4.0) _e_		Comparison long to short
			FLTF Long leg		60.8^d^ (292) _e_	BF **p = 0.022;**
			FLTF Short leg		60.0^d^ (4.16) _e_	FLTF p=0.241;
			SSF Long leg		62.5^d^ (1.91) _e_	SSF **p=0.005**
			SSF Short leg		58.9^d^ (3.90) _e_	
Stride length (m)			BF	1.12^d^ (0.13) _e_		Friedman ANOVA: **p<0.05**
			FLTF		1.24^d^ (0.12) _e_	Post hoc:
			SSF		1.24^d^ (0.12) _e_	BF vs. FLTF **p<0.05;**
						BF vs. SSF **p<0.05**
Velocity (m/s)			BF	1.18^d^ (0.16) _e_		Friedman ANOVA: **p<0.05**
			FLTF		1.24^d^ (0.12) _e_	Post hoc:
			SSF		1.21^d^ (0.22) _e_	BF vs. FLTF **p<0.05**

*AFO* Ankle Foot Orthosis, *BF* Barefoot, *CoP* Centre of Pressure, *CTEV* Congenital Talipes Equino Varus, *CTF* Corrective Therapeutic Footwear, *DB* Denis Brown Barred Night Boot, *FAS* Forefoot Abduct Night Shoe, *FITF* Functional Instability Therapeutic Footwear, FLTF Functional Lift Therapeutic Footwear, *FO* Foot Orthoses, *FSTF* Functional Stability Therapeutic Footwear, *N/A* Not Applicable, *SLF* Standard Last Footwear, *SSF* Standard Sole Footwear, ^a^ supplementary results in additional file 3, ^b^ 95% Confidence Interval, ^d^ Median, ^e^ Inter Quartile Range,

**Table 5:** Secondary outcome measures

Outcome	Study	Condition	Group	Baseline Mean (SD ±/-)	Final^**a**^ Mean (SD +/-)
**Functional Stability Therapeutic Footwear**					
Pain whilst using device	Bakker et al. (1997) **[**38**]**	DMD	FSTF	N/A	1.42 (0.53)
1=no pain			Own footwear	N/A	1.02(0.51)
			KAFO	N/A	3.0 (1.87)
5=great deal of pain			SF	N/A	2.33 (1.03)
			AFO	N/A	2.20 (1.39)
Reluctance to use device			FSTF	N/A	2.28 (1.25)
1=not reluctant			Own footwear	N/A	1.29 (1.35)
			KAFO	N/A	3.0 (1.58)
5=great deal of reluctance			SF	N/A	3.66 (1.21)
			AFO	N/A	2.85 (1.53)

*AFO* Ankle Foot Orthosis, *DMD* Duchenne Muscular Dystrophy, *FSTF* Functional Stability Therapeutic Footwear, *KAFO* Knee Ankle Foot Orthosis, *N/A* Not Applicable, *SF* Standing Frame, ^a^No statistical test for significance performed

Analysis and synthesis of the studies were performed according to the grouping/subgrouping of footwear interventions, with further subdivision by the medical condition of the study participants (see Table 1).

### Therapeutic footwear interventions

The types of therapeutic footwear interventions that were explored in the 13 studies fell into 2 of the previously defined groupings from the scoping review [24] corrective (n=3) and functional (n=10) (Table 1). No studies explored the effects of accommodative therapeutic footwear for children. None of the included studies reported adverse events or stated if such events were considered within the study plan.

#### Corrective Footwear

These three studies focused on the effects of the footwear on lower limb alignment pes planus (n=2) [12, 37] and congenital talipes equino varus (CTEV) (n=1 )[16] (Table 1). The studies were all randomised controlled trials (level II evidence). Two of the studies were of fair QI [16, 37] and one of poor QI [12]. A total of 196 children were examined across the studies with an age range from 11 months to 5 years (Table 1, Additional file 2). One study failed to report the sex distribution amongst the experimental groups [12], and the height and mass of the participants were only reported in one study [16]. Two of the studies [16, 37] had sufficient sample size to detect a medium effect size of 0.3 at 0.05 significance and 80% power [45]. However, one of the studies suffered a loss to follow up >20% [37] with no intention to treat factored into the analysis.

Various design characteristics were reported for the corrective footwear (Table 2) in the three studies. Consistent features appeared to be some form of reinforced or lengthened heel counter or arch inlay [12, 16, 37]. The common comparator to corrective footwear interventions across all three studies was daily wear of standard retail footwear (see Table 1). One study also considered orthotic arch support or heel cups [37]. Assessors were blinded in only one of the three studies [37]. Primary outcomes focused on skeletal geometric measures which were presented in the three studies included in this grouping (Table 3). These were radiographic measures of the skeletal alignment of the foot in two studies considering pes planus [12, 37], and 3D scanned images of the foot and ankle for the study considering CTEV [16]. Only one study in this grouping [16] considered biomechanical outcomes (Table 4) consisting of pressure ratios of the heel to forefoot and heel to lateral midfoot in walking conditions. Secondary outcomes, as determined by this current review, were not reported in any study amongst the corrective footwear grouping. Results indicated that there was no statistically significant effect of corrective footwear versus readily available retail footwear in the developmental of asymptomatic paediatric pes planus. Daily wear of corrective footwear in combination with nocturnal wear of Dennis Brown splint did not appear to offer any difference to the 3D scans of the trans-malleolar axis, and the bean-shaped ratio of CTEV in comparison to daily wear of standard footwear and nocturnal wear of Dennis Brown splint [16]. However, the study did demonstrate statistically significant improvements in 4 of the 13 plantar pressure measures (Table 4) indicating a reduction of equinus and varus deformity with the daily wear of corrective footwear and nocturnal use of Dennis Brown splint. Results for the nine plantar pressure measures that were not statistically significant concerning CTEV and corrective footwear, but highlighted the effects of different nocturnal splints, can be found in Additional file 3.

#### Functional Footwear

Functional footwear intervention studies focused chiefly on biomechanical primary outcomes (kinematic, kinetic, spatiotemporal, balance) (Table 4, Additional file 3) which were considered in 7 of the 10 studies [10, 11, 14, 40–43]. Skeletal geometry primary outcomes (Table 3) were considered in only two of the studies [39, 44]. Secondary outcomes were considered in two studies [38, 39] but empirically reported in one [38] (Table 5). A total of 311 children were considered amongst the studies with an age range from 3 to 17 years (Table 1, Additional file 2). Reporting of the participants’ height and mass was provided in four studies [10, 11, 14, 43] (Table 1). It was noted that the small sample size affected the statistical power in all but two of the experimental studies amongst the functional footwear grouping studies [11, 46]. None of the studies blinded the participants to the intervention, with only one study blinding the assessor [14]. Sufficient information on the participant recruitment strategy was provided in only two studies [10, 38]. Three of the studies stipulated a brief wearing in period to allow the child to become accustomed to walking in the interventions [10, 11, 14]. Functional footwear was split into three design characteristic subgroups: Stability, Instability, and Lift as defined by the previous scoping review [24]**.**

##### Stability footwear

There were seven studies in this subgrouping with various footwear designs used amongst the studies (Table 2). Five studies involved footwear offering some form of medial-lateral stability with arch inlay and/or reinforced heel counte r[11, 14, 38, 39, 41] and two studies involved footwear with anterior-posterior sole wedging that work alongside Ankle Foot Orthoses (AFO) to offer sagittal stability [40, 42]

In relation to footwear that offered mediolateral stability, the study designs consisted of four before-after studies (level III evidence) [11, 14, 39, 41] and one survey study (level IV evidence) [38]. Two of these studies were of fair QI [11, 14] and two poor QI [39, 41]. The survey study met 64% of the survey quality criteria [38]. The medical conditions of the participants were mobile pes planus, Down’s syndrome, in-toeing and Duchenne muscular-dystrophy (Table 1) [11, 14, 38, 39, 41]. Various comparators were considered (Table 1). Barefoot conditions, walking or stance, was the baseline assessment in three of the five studies [11, 14, 39]. Standard unmodified footwear was considered in three of the studies [38, 39, 41]. Arch inlays/foot orthosis was a comparator either fitted to stability footwear in one paper [39] or to standard footwear in another paper [11]. AFOs, Knee Ankle Foot Orthoses (KAFO), and standing frames were additionally considered in one study [38]. Medical taping was a consideration in one study [14]. Stability footwear with various sole modifications were compared in one study [41].

Primary outcomes considered both biomechanical (Table 4) and skeletal geometric measures (Table 3). One study demonstrated statistically significant changes in spatiotemporal parameters (increase in velocity and stride length, reduction in the base of support) in children with Down’s syndrome whilst wearing stability footwear compared to the barefoot condition [14]. However, no differences were noted between the stability footwear group and the taping comparator group in this study [14]. One study demonstrated a statistically significant reduction in the centre of pressure (CoP) displacement and increased step velocity in the stability footwear compared to the barefoot condition for individuals with pes planus [20]. No statistically significant difference was found in step symmetry in this study between barefoot and stability footwear conditions. However, the regular shoe with orthosis demonstrated a statistically significant increase in step symmetry compared to stability footwear conditions [11]. Mediolateral wedged sole modifications were shown to have no statistically significant effect on in-toed angle of gait. Torqheels (circular sole additions that impart a torque on ground contact [47]) did show a statistically significant reduction of the in-toed angle of gait (approximately 33%) compared to a standard soled footwear [41]. Skeletal geometry outcomes used were immediate weight-bearing radiographic alignment changes to the medial longitudinal arch in participants with symptomatic pes planus. Skeletal alignment was seen to improve in stability footwear vs. barefoot conditions [39]. However, no statistical analysis was performed on these effects. Additionally, there was absent reporting of the changes to these angles in standard footwear conditions [39].

Secondary outcomes, as outlined by this review, were explored in two of the mediolateral stability footwear studies. Reduction of foot fatigue and pain in pes planus were investigated in one paper [39]. This paper demonstrated these outcomes improved for the stability footwear intervention compared to standard footwear and arch inlay. However, no statistical analysis was performed on these findings. The second paper considered surveying parents of children with Duchenne’s muscular dystrophy (DMD) as to the reluctance to use the prescribed assistive device and pain whilst using the device [38] (Table 5). This demonstrated that stability footwear was associated with less reluctance to wear and less pain experienced compared to AFOs, KAFOs and standing frames. This study failed to provide information on the design or testing of the questionnaire. Additionally, there was no statistical analysis performed, and it was unclear as to the severity of the DMD amongst the different interventions or if the pain measured was from the device or from the condition itself.

In relation to footwear that offered sagittal stability, study design consisted of one before-after design [40] and one cross over study [42]; both studies were of fair QI. The medical conditions of the participants consisted of spastic cerebral palsy with knee hyperextension [40] or crouch gait [42] (Table 1). Comparators consisted of standard footwear in one study [42] and AFO worn with standard footwear combination in both studies [40, 42] (Table 1). Biomechanical outcomes were considered in both these studies [40, 42] (Table 4). One study demonstrated a statistically significant improvement on knee hyperextension and shank to vertical angle (SVA) kinematics in sagittal wedged soled footwear in combination with AFO compared to the standard sole footwear with AFO [40]. However, no kinetic or spatiotemporal variable reached statistical significance [40]. Standing balance was considered in the second study. This study found statistically significant improvement for differences in standing balance in a sub-set analysis of diplegic individuals with Gross Motor Function Scores (GMFS) 2-3 for AFOs and anteroposterior sagittal wedged footwear combination intervention compared to both standard footwear and AFO standard footwear combination [42].

##### Instability footwear

This subgrouping consisted of one study that considered commercially available MBT® footwear [43]. This footwear consists of a rounded sole shoe with a midfoot pivot [43] (Table 2). The study was a before-after design (level III evidence) of poor QI. The health conditions considered were highly varied in the group and consisted of cerebral palsy, Prader Willi, unspecified motor and development delay, Cornelia de Lange syndrome and Attention Deficit Hyperactive Disorder (ADHD) (Table 1). The grading and degree of the mobility impairments of the participants were not fully described. All individuals wore the MBT footwear for the 8-week period and were tested barefoot and in the MBT footwear (Table 1 and 4). Biomechanical outcomes of static and dynamic balance were considered in this study (Table 4). No spatiotemporal kinematic or kinetic outcomes were considered. This footwear did not demonstrate any statistically significant effects on static balance and a number of dynamic balance outcomes; these results are presented in Additional file 3. However, two of the dynamic balance outcomes were seen to statistically significantly improve, with a reduction in the number of falls seen over the course of the study and improvement in the mediolateral control of the centre of pressure displacement. It must be noted that two of the participants were unavailable for the four-week testing point and one other participant failed to understand the instructions for the control aspect of dynamic balance testing. Intention to treat analysis was not reported to account for this drop off in participation.

##### Lift footwear

Lift footwear was described as consisting of unilateral outer-sole adhesions (Table 2) [10, 44]. This subgroup consisted of two before and after studies; one fair QI [10] and one poor QI [44]. Poor reporting of the intervention and the participants affected the QI of one study [44]. Conditions considered were limb length inequality in combination with either, idiopathic scoliosis [44] or spastic cerebral palsy [10] (Table 1). Barefoot conditions, walking or stance, was considered as a comparator in both studies [10, 44], with standard sole footwear also considered in one study [10] (Table 1). Spatiotemporal and kinematic variables were considered in individuals with spastic cerebral palsy and limb length inequality, in one of the studies [10] (Table 4). Statistically significant differences seen between stance time in the long and short leg in barefoot and unmodified shod conditions were not seen in the lifted footwear intervention. Velocity was also statistically significantly increased in the lifted footwear compared to the barefoot conditions. Statistically significant kinematic differences between hip flexion at initial contact and swing and ankle dorsiflexion in stance seen between the long and short limb in the barefoot condition were no longer significant in the lifted footwear condition. The second study considered skeletal geometric measures of acute changes on, lower limb, pelvic, and spinal posture through radiographs and 3D marker system of first barefoot, then lifted sole conditions [44] (Table 3). Sole lifted conditions statistically significantly reduced the Cobb angle, pelvic tilt, version between right and left iliac bones, and shoulder tilt compared to barefoot conditions. These findings are thought to demonstrate acute improvements in idiopathic scoliosis posture.

## Discussion

The review identified 13 empirical studies that explored the effects of therapeutic footwear in children with mobility impairment. Study quality was negatively affected in most studies by the reporting strategy, with a lack of descriptions of basic participant anthropometrics and inadequate blinding of participants and assessors impacting on generalisability and internal and external validity. Another consideration that may impact on long term conservative footwear management is compliance with the intervention [48]; this was not accounted for or was inconsistently measured in the studies potentially introducing confounding bias [49, 50].

The medical conditions with the highest number of studies were pes planus (five studies) and cerebral palsy (four studies). It must be noted three studies, considering pes planus appeared to only acknowledge the postural presentation with no apparent symptoms or other underlying pathology identified [11, 12, 37]. Therapeutic interventional studies should consider expanding on the descriptors of inclusion for pes planus in children [51–53] to avoid the possibility of medicalising healthy physiological development [54] and potential detriment to the health economy and the individual [55, 56]. The effects of footwear as a therapeutic intervention on other noteworthy conditions that impact on children’s mobility such as joint hypermobility syndrome [57], spina bifida [58], developmental coordination disorder [59], juvenile idiopathic arthritis [60], and Charcot Marie Tooth [61] were not considered in the included studies.

The age of the participants showed distinct differences between the two main footwear groupings with corrective footwear considering a younger age range (11 months to 5 years) and functional footwear a broader age range (3 to 15 years) (Additional file 2). This may be explained by the increased percentage of cartilage in the infant skeleton having the perceived potential to be influenced by conservative intervention [62, 63] in relation to corrective footwear, and the broader age range in functional footwear linked to the ongoing need for assistive aid for children with mobility issues in daily activity. Primary outcomes were focused towards skeletal geometry in all of the corrective footwear studies as would be expected since the aim of treatment is to bring about realignment of the skeletal system in the lower limb [24]. Primary outcomes for functional footwear were focused on biomechanical variables in 7 out of the 10 studies. This again would be expected since the purpose of functional footwear is to assist children’s gait parameters [24].

In consideration of corrective footwear grouping**,** the studies explored their effects for asymptomatic flexible pes planus and CTEV alongside nocturnal barred footwear post serial casting. One fair quality study, for a relatively large sample size, would suggest that corrective footwear offers no effect on mobile asymptomatic pes planus in children [37]. One fair quality controlled group study [16] suggests daily use of corrective footwear alongside nocturnal splinted footwear can improve the equinus and varus positioning of the forefoot. However, caution must be observed as CTEV is a heterogeneous pathology [64], and this study failed to report the aetiologies of the participants’ deformities, thus affecting the generalisability of the study’s findings.

The studies across the subgroupings of functional footwear were mainly experimental before and after design and one survey (Additional file 4 and 5). The significance of the changes observed in these studies could have been a short term effect [65] due to an insufficient wearing in and accommodation period. A learning effect could also impact on the findings [66] with participants able to anticipate factors such as those that required dynamic balance [43]. Further research with suitable wearing in periods and a control group study design would be beneficial to corroborate the findings of these studies. Stability footwear was seen to comprise of two general designs; one to assist mediolateral stability [11, 14] and one to work alongside AFOs to assist sagittal stability [40, 42]. For mediolateral stability design, one fair quality study demonstrated statistically significant effects on velocity and mediolateral CoP displacement in children with pes planus between stability footwear intervention and barefoot [11] with one further fair quality study demonstrating statistically significant effects on velocity, stride length, and base of support for stability footwear vs. barefoot conditions in individuals with Down’s syndrome [14]. However, both the studies did not compare these effects with a standard children’s footwear condition that has also demonstrated statistically significant effects on spatiotemporal measures in children compared to barefoot conditions [18]. This opens the significance of the spatiotemporal findings for this footwear to debate and precludes any recommendations advocating this intervention over standard footwear in clinical practice for children with flexible pes planus or Downs syndrome. There is poor quality evidence that sole modification reduces the in-toed angle of gait by a third compared to standard sole footwear; however, the need to treat this developmental variant conservatively is debatable [67]. One survey indicated that stability footwear was associated with less reluctance to use and less pain than other assistive devices in individuals with DMD. However, the severity of the condition amongst those using the various devices was not stated; this precluded any informed clinical recommendation for the use of stability footwear in this condition. Those studies that considered sagittal stability demonstrated fair quality evidence in two studies that this footwear combined with a customised AFO can improve, knee and shank vertical angle in spastic CP [40] or standing balance in spastic diplegic GMFS 2-3 crouch gait [42]. Therefore sagittal stability footwear could tentatively be recommended over standard retail footwear for AFO footwear combination in children with spastic CP. Evidence indicated that instability footwear improves dynamic balance (number of falls and control of mediolateral CoP displacement) in a range of children’s developmental disabilities. However, the quality of this study was poor, with failure to account for dropout across the testing period, and a diverse range of mobility impairments considered in the sample (Table 1) questioning the validity of the central trend analysis obtained [43].

Lift footwear offered fair quality evidence in one study to improve the symmetry of a wide range of kinematic and spatiotemporal gait parameters between the long and short limb in individuals with spastic cerebral palsy potentially supporting its use for individuals with this clinical presentation [10]. Spinal and pelvic skeletal geometry were seen to improve in individuals with idiopathic scoliosis; however, this was of poor quality with no standard footwear comparator and insufficient information provided on the participants and recruitment strategy opening the significance and generalisability of the findings to debate [44].

It was noted that a number of studies amongst the functional footwear grouping contained a degree of heterogeneity in the participant’s age ranges and variable motor impairment. There were over seven year age ranges in some studies [40, 42, 43]; since development affects biomechanical parameters [18, 68], this should be considered when averaging biomechanical outcome data. Further consideration should be given to the studies that averaged biomechanical outcome data amongst individuals with cerebral palsy [10, 40, 42] as this condition has a significant range of motor impairment that may not be amenable to central trend analysis [69–72].

There is relatively limited research concerning any grouping of therapeutic footwear. Level of evidence ranged from II to IV, but no study exceeded a quality assessment of fair, due to methodology that affected both internal and external validity. This entails a conservative recommendation from the current evidence base concerning clinical usage of therapeutic footwear. There appears to be evidence that corrective footwear is not recommended as an intervention for developmental pes planus since there is no apparent favourable outcome compared to standard footwear in infants and young children. With an unnecessary prescription of corrective footwear leading to potential over-medicalisation of typical development and psychosocial detriment in early adult life [54, 56]. Functional footwear appears to be able to play a role in assisting children with mobility impairment across a broader age range than corrective footwear; however, these studies invariably suffer from a small sample size potentially being underpowered to detect any statistically significant effect. Future studies for functional therapeutic footwear must consider a comparison with standard footwear, as suggested by Wegener et al. [18], to factor in the effects of regular footwear on children’s gait in comparison to barefoot conditions. Further comparison to other assistive devices such as foot orthoses is warranted in order to inform when stability footwear should be used as an alternative or in combination with foot orthoses, and where lift therapy for limb length inequality is best addressed with removable inlays or external shoe modifications.

Other recommendations for general therapeutic footwear research include clear reporting of participant characteristics and the distribution of demographics between treatment groups, to include, sex, height and mass which have demonstrated effects on foot function and skeletal geometry in previous studies [73–75]. Consideration of participant recruitment strategies is required; being mindful of institutional bias in the samples selected, and more transparent recruitment reporting to inform on the external validity of the work [33]. The lack of consideration of adverse events across the studies warrants comment since it is imperative intervention studies declare adverse events or the measures taken to capture these, as appropriate evidence base should identify the potential harms as well as benefits of any therapeutic intervention [76].

The psychosocial impact of therapeutic intervention is an important consideration for mobility-impaired children [77]. The World Health Organisation’s international classification of function for children living with disabilities considers a number of factors to ensure the child can achieve the highest quality of life [2]. The current evidence base concerning therapeutic footwear has chiefly focused on the body structure and functional aspects of the ICF-CY but has not attempted to assess the long-term or psychosocial effects the intervention may have on the child’s quality of life in terms of the ability to participate in daily activities or relief of pain.

### Limitations of the current study

﻿The initial screening of the studies that identified children’s therapeutic footwear was performed independently by the one author (MH) during the preceding scoping review [24], which may have opened these processes to personal bias. The review has considered only those articles with an available English language abstract which may have impacted on the scope of research. Incomplete description of the therapeutic footwear together with the lack of information on basic anthropometrics (height, mass, BMI), heterogeneity of the participants, and the broad range of outcomes precluded a quantitative analysis of the aggregated results which could be perceived as a limitation. There were 76 different outcome measures considered across the included studies with few reporting on the same outcome measures. The definition and adoption by researchers to minimum sets of condition-specific outcome measures, such as those presented by the International Consortium for Health Outcome Measurement (ICHOM) [78], will enable between study comparisons and meta-analyses of future research.

## Conclusion

There are a limited number of studies exploring the effects of children’s therapeutic footwear; these have mainly been studied on children with pes planus and cerebral palsy. Limited fair quality level II evidence is available that corrective footwear has no statistically significant effect on apparent typical developmental pes planus. Conversely, there is limited fair quality level II evidence that it can offer a corrective effect in mild to moderate cases of CTEV in infancy. Functional therapeutic footwear offers limited fair quality level III evidence on apparent improvement to gait parameters in pre-school and primary school-aged children with pes planus, Down’s syndrome or CP. Included studies explored body structure and functional aspects of the WHO ICF-CY (biomechanical and skeletal geometry outcomes). However, psychosocial aspects of the ICF-CY concerning the quality of life appears largely absent in the research.

Review findings suggest that further research on therapeutic footwear with robust study designs is warranted. The outcome measures should consider a full range of ICF-CY aspects, and the reporting should include a clear description of the footwear interventions, participant characteristics, recruitment strategy and measures of adverse events. These recommendations will improve the current evidence base for therapeutic footwear as an intervention for children with mobility impairment.

## Supplementary information

**Additional file 1.** Example of Medline (EBSCO) search strategy

**Additional file 2.** Age ranges for children with mobility impairment in the included studies.

**Additional file 3.** Supplementary biomechanical outcome results

**Additional file 4.** Evidence level and quality assessment of experimental studies

**Additional file 5.** Level of evidence and quality assessment of survey study.

**Additional file 6.** PRISMA 2009 Checklist.

## Data Availability

All data generated or analysed during this study are included in this published article [and its additional files].

## Abbreviations

AFO: Ankle foot orthoses
CoP: Centre of pressure
CTEV: Congenital talipes equino varus
DMD: Duchenne’s muscular dystrophy
GMFS: Gross Motor Function Scores
KAFO: Knee Ankle Foot Orthoses
OCEBM: Oxford Centre for Evidence-Based Medicine
QI: Quality assessment index
RCTs: Randomised control trials
WHO ICF-CY: World Health Organisation’s international classification of function child and youth version
