# Supplementary material for: Effectiveness of therapeutic footwear for children: A systematic review
Source: J Foot Ankle Res. 2020 May 13;13:23. doi: 10.1186/s13047-020-00390-3 (PMC7222438; doi:10.1186/s13047-020-00390-3)
Supplement: Supplementary file 2 — Additional file 2. Age ranges for children with mobility impairment in the included studies. [file 13047_2020_390_MOESM2_ESM.docx]

Additional File 2

Corrective

Age ranges for children with mobility impairment in the included studies.

*Mean (+/- SD), † Age when tested, ‡ Age at entry of study
