# Supplementary material for: Effectiveness of therapeutic footwear for children: A systematic review
Source: J Foot Ankle Res. 2020 May 13;13:23. doi: 10.1186/s13047-020-00390-3 (PMC7222438; doi:10.1186/s13047-020-00390-3)
Supplement: Supplementary file 3 — Additional file 3. Supplementary biomechanical outcome results [file 13047_2020_390_MOESM3_ESM.docx]

Additional File 3: Supplementary biomechanical outcome results

| **Outcome** | **Study** | **Condition** | **Group** | **Baseline**  **Mean (SD +/-)** | **Final**  **Mean (SD +/-)** | **Statistical Result**  **(Significant values given in bold)** |
| --- | --- | --- | --- | --- | --- | --- |
| **Corrective Therapeutic Footwear** | | | | | | |
| **Biomechanical outcomes** | | | | | | |
| **Plantar pressure** | | | | | | |
| **Average peak pressure (kPa): Hindfoot** | **Chen et al. (2015)** | CTEV | Group 1 CTF and DB | N/A | 74.1 (64.02-84.18) * | One-way MANOVA:  **p=0.024** |
|  |  |  | Group2 DB and Own footwear | N/A | 57.48 (39.47-75.49) * | Post hoc:  Group 2 vs. Group 3 **p<0.05** § |
|  |  |  | Group 3 FAS and CTF | N/A | 83.18 (71.78-94.58) * |  |
| **Average peak pressure (kPa): Medial midfoot** |  |  | Group 1 CTF and DB | N/A | 55.51 (41.82-69.21) * | One-way MANOVA:  p>0.05 |
|  |  |  | Group2 DB and Own footwear | N/A | 59.58 (43.14-76.01) * |  |
|  |  |  | Group 3 FAS and CTF | N/A | 47.5 (41.20-53.80) * |  |
| **Average peak pressure (kPa): Lateral forefoot** |  |  | Group 1 CTF and DB | N/A | 55.44 (46.02-64.87) * | One-way MANOVA:  p>0.05 |
|  |  |  | Group2 DB and Own footwear | N/A | 66.09 (50.02-82.15) * |  |
|  |  |  | Group 3 FAS and CTF | N/A | 55.15 (42.37-67.94) * |  |
| **Average peak pressure (kPa): Medial forefoot** |  |  | Group 1 CTF and DB | N/A | 95.54 (83.89-107.19) * | One-way MANOVA:  **p=0.049** |
|  |  |  | Group2 DB and Own footwear | N/A | 89.34 (66.31-112.33) | Post hoc:  Group 2 vs. Group 3 **p<0.05** § |
|  |  |  | Group 3 FAS and CTF | N/A | 122.58 (100.78-124.38) * |  |
| **Maximum peak pressure (kPa): Lateral midfoot** |  |  | Group 1 CTF and DB | N/A | 99.14 (89.06-109.22) * | One-way MANOVA:  **p=0.033** |
|  |  |  | Group2 DB and Own footwear | N/A | 105.89 (84.27-127.52) * | Post hoc:  Group 1 vs. Group 3 **p<0.05** §  Group 2 vs. Group 3 **p<0.05** § |
|  |  |  | Group 3 FAS and CTF | N/A | 82.38 (71.87-92.90) * |  |
| **Maximum peak pressure (kPa): Medial midfoot** |  |  | Group 1 CTF and DB | N/A | 63.69 (51.88-75.50) * | One-way MANOVA:  p>0.05 |
|  |  |  | Group2 DB and Own footwear | N/A | 56.8 (45.64-67.96) * |  |
|  |  |  | Group 3 FAS and CTF | N/A | 56.44 (46.69-66.20) * |  |
| **Maximum peak pressure (kPa): Lateral forefoot** |  |  | Group 1 CTF and DB | N/A | 118.48 (105.96-131) * | One-way MANOVA:  p>0.05 |
|  |  |  | Group2 DB and Own footwear | N/A | 120.53 (104.55-136.51) |  |
|  |  |  | Group 3 FAS and CTF | N/A | 129.77 (112.98-146.55) * |  |
| **Maximum peak pressure (kPa): Medial forefoot** |  |  | Group 1 CTF and DB | N/A | 115 (101.83-128.16) * | One-way MANOVA:  **p=0.008** |
|  |  |  | Group2 DB and Own footwear | N/A | 101.26 (81.02-121.51) * | Post hoc:  Group 1 vs. Group 3 **p<0.05** §  Group 2 vs. Group 3 **p<0.01** § |
|  |  |  | Group 3 FAS and CTF | N/A | 135.87 (122.1-149.64) * |  |
| **Peak pressure ratio: Medial/lateral forefoot** |  |  | Group 1 CTF and DB | N/A | 1.52 (1.28-1.76) * | One-way MANOVA:  p>0.05 |
|  |  |  | Group2 DB and Own footwear | N/A | 1.37 (0.96-1.78) * |  |
|  |  |  | Group 3 FAS and CTF | N/A | 1.52 (1.28-1.75) * |  |
| **Functional Instability Therapeutic Footwear** | | | | | | |
| **Biomechanical outcomes** | | | | | | |
| **Balance (Dynamic)** | | | | | | |
| **Anterior posterior control (CoP)** | **Ramstrand et al. (2008)** | Cerebral Palsy + mixed developmental disability | BF Slow | 51 (33.9-68.1) * |  | BF p>0.05; FITF p>0.05 |
|  |  |  | BF Medium | 40.67 (15.9-65.5) * |  |  |
|  |  |  | BF Fast | 35.6 (11.6-59.7) * |  |  |
|  |  |  | FITF Slow | 33.78 (12.8-54.8) * |  |  |
|  |  |  | FITF Medium | 31.44 (7.1-55.8) * |  |  |
|  |  |  | FITF Fast | 36.89 (13.5-60.3) * |  |  |
|  |  |  | BF Slow (at 4 weeks) |  | 43 (19.9-66.6) * |  |
|  |  |  | BF Fast (at 4 weeks) |  | 53.9 (41.9-66.4) * |  |
|  |  |  | FITF Slow (at 4 weeks) |  | 30.67 (8.2-53.1) * |  |
|  |  |  | FITF Fast (at 4 weeks) |  | 52.11 (30.3-74.0) * |  |
|  |  |  | BF Slow (at 8 weeks) |  | 51.6 (36.9-66.1) * |  |
|  |  |  | BF Medium (at 8 weeks) |  | 62.67 (45.7-79.7) * |  |
|  |  |  | BF Fast (at 8 weeks) |  | 56.89 (36.1-77.7) * |  |
|  |  |  | FITF Slow (at 8 weeks) |  | 55.44 (41.4-69.5) * |  |
|  |  |  | FITF Medium (at 8 weeks) |  | 40.44 (18.0-62.9) * |  |
|  |  |  | FITF Fast (at 8 weeks) |  | 51.78 (30.5-73.1) * |  |
| **Number of falls toes down condition** |  |  | Subjects 1,2,9 | 0 |  | Unable to test, requirements for statistical test violated |
|  |  |  | Subject 3 | 7 |  |  |
|  |  |  | Subjects 4,10 | 2 |  |  |
|  |  |  | Subject 5 | 1 |  |  |
|  |  |  | Subject 6 | 5 |  |  |
|  |  |  | Subject 7,8 | 9 |  |  |
|  |  |  | Subject 1 (at 4 weeks) |  | 1 |  |
|  |  |  | Subject 2, 6 (at 4 weeks) |  | Did not participate |  |
|  |  |  | Subject 3 (at 4 weeks) |  | 6 |  |
|  |  |  | Subject 4 (at 4 weeks) |  | 8 |  |
|  |  |  | Subject 5 (at 4 weeks) |  | 4 |  |
|  |  |  | Subject 7 (at 4 weeks) |  | 7 |  |
|  |  |  | Subject 8,9,10 (at 4 weeks) |  | 0 |  |
|  |  |  | Subjects 1,2,4,5,8,9,10 (at 8 weeks) |  | 0 |  |
|  |  |  | Subject 3 (at 8 weeks) |  | 3 |  |
|  |  |  | Subject 6 (at 8 weeks) |  | 2 |  |
|  |  |  | Subject 7 (at 8 weeks) |  | 1 |  |
| **Balance (Static)** | | | | | | |
| **Frequency Hz (revolutions of CoP)** | Ramstrand et al. (2008) | Cerebral Palsy + mixed developmental disability | BF Eyes open | 0.79 (0.7-0.9) * |  | Friedman ANOVA:  BF p>0.05; FITF p>0.05 |
|  |  |  | FITF Eyes open | 0.85 (0.7-1) * |  | Wilcoxon signed rank  BF vs. FITF p>0.05 |
|  |  |  | BF Eyes closed | 0.7 (0.5-0.9) * |  |  |
|  |  |  | FITF Eyes closed | 0.61 (0.5-0.7) * |  |  |
|  |  |  | BF Eyes open (at 4 weeks) |  | 0.68 (0.6-0.8) * |  |
|  |  |  | FITF Eyes open (at 4 weeks) |  | 0.68 (0.6-0.8) * |  |
|  |  |  | BF Eyes closed (at 4 weeks) |  | 0.66 (0.6-0.8) * |  |
|  |  |  | FITF Eyes closed (at 4 weeks) |  | 0.62 (0.5-0.7) * |  |
|  |  |  | BF Eyes open (at 8 weeks) |  | 0.69 (0.6-0.8) * |  |
|  |  |  | FITF Eyes open (at 8 weeks) |  | 0.72 (0.6-0.8) * |  |
|  |  |  | BF Eyes closed (at 8 weeks) |  | 0.71 (0.6-0.8) * |  |
|  |  |  | FITF Eyes closed (at 8 weeks) |  | 0.67 (0.6-0.8) * |  |
| **Path length (cm/sec) (CoP)** |  |  | BF Eyes open | 2.66 (2.3-3.0)* |  | Friedman ANOVA:  BF p>0.05; FITF p>0.05  Across testing occasions |
|  |  |  | FITF Eyes open | 3.94 3.3-4.6)* |  | Wilcoxon signed rank  BF vs. FITF **p<0.05**  Across testing occasions |
|  |  |  | BF Eyes closed | 3.28 (2.8-3.7)* |  |  |
|  |  |  | FITF Eyes closed | 5.82 (4.5-7.1)* |  |  |
|  |  |  | BF Eyes open (at 4 weeks) |  | 2.56 (2.1-3.0)* |  |
|  |  |  | FITF Eyes open (at 4 weeks) |  | 3.64 (2.7-4.5)* |  |
|  |  |  | BF Eyes closed (at 4 weeks) |  | 3.04 (2.3-3.7)* |  |
|  |  |  | FITF Eyes closed (at 4 weeks) |  | 4.51 (3.8-5.3)* |  |
|  |  |  | BF Eyes open (at 8 weeks) |  | 2.63 (2.2-3.0)* |  |
|  |  |  | FITF Eyes open (at 8 weeks) |  | 4.14 (5.0-4.1)* |  |
|  |  |  | BF Eyes closed (at 8 weeks) |  | 3.29 (2.8-3.7)* |  |
|  |  |  | FITF Eyes closed (at 8 weeks) |  | 4.7 (4.1-5.4) * |  |
| **Radial displacement (cm) (CoP)** |  |  | BF Eyes open | 0.59 (0.5-0.7)* |  | Friedman ANOVA:  p>0.05; FITF p>0.05  Across testing occasions |
|  |  |  | FITF Eyes open | 0.86 (0.6-1.1)* |  | Wilcoxon signed rank  BF vs. FITF **p<0.05**  Across testing occasions |
|  |  |  | BF Eyes closed | 0.85 (0.7-1.0)* |  |  |
|  |  |  | FITF Eyes closed | 1.56 (1.2-1.9)* |  |  |
|  |  |  | BF Eyes open (at 4 weeks) |  | 0.64 (0.5-0.8)* |  |
|  |  |  | FITF Eyes open (at 4 weeks) |  | 0.92 (0.6-1.2)* |  |
|  |  |  | BF Eyes closed (at 4 weeks) |  | 0.79 (0.6-1)* |  |
|  |  |  | FITF Eyes closed (at 4 weeks) |  | 1.22 (0.9-1.5) * |  |
|  |  |  | BF Eyes open (at 8 weeks) |  | 0.67 (0.5-0.9)* |  |
|  |  |  | FITF Eyes open (at 8 weeks) |  | 0.99 (0.7-1.3)* |  |
|  |  |  | BF Eyes closed (at 8 weeks) |  | 0.83 (0.6-1.1)* |  |
|  |  |  | FITF Eyes closed (at 8 weeks) |  | 1.28 (0.9-1.6)* |  |

**AFO** Ankle Foot Orthosis, **BF** Barefoot, **CoP** Centre of Pressure, **CTEV** Congenital Talipes Equino Varus, **CTF** Corrective Therapeutic Footwear, **DB** Denis Brown Barred Night Boot, **FAS** Forefoot Abduct Night Shoe, **FITF** Functional Instability Therapeutic Footwear, **FLTF** Functional Lift Therapeutic Footwear, **FO** Foot Orthoses, **FSTF** Functional Stability Therapeutic Footwear, **N/A** Not Applicable, **SLF** Standard Last Footwear, **SSF** Standard Sole Footwear, ***** 95% Confidence Interval, **†** Median, **‡** Inter Quartile Range § Post Hoc Test
