# Supplementary material for: Effectiveness of therapeutic footwear for children: A systematic review
Source: J Foot Ankle Res. 2020 May 13;13:23. doi: 10.1186/s13047-020-00390-3 (PMC7222438; doi:10.1186/s13047-020-00390-3)
Supplement: Supplementary file 4 — Additional file 4. Evidence level and quality assessment of experimental studies [file 13047_2020_390_MOESM4_ESM.docx]

Additional File 4 : Evidence level and quality assessment of experimental studies

| **Author Year** | **Level of Evidence**  **OCEBM**  **Study Design** | **Quality Assessment, Modified Downs and Black** | | | | | | | | | | | | | | | | | | | | | | | | | | | |
| --- | --- | --- | --- | --- | --- | --- | --- | --- | --- | --- | --- | --- | --- | --- | --- | --- | --- | --- | --- | --- | --- | --- | --- | --- | --- | --- | --- | --- | --- |
|  |  | **Reporting** | | | | | | | | | | **External validity** | | | **Internal validity bias** | | | | | | | **Internal Validity-Confounding (Selection bias)** | | | | | | **Power** | **Total**  **Score**  **% Score** |
|  |  | **1** | **2** | **3** | **4** | **5** | **6** | **7** | **8** | **9** | **10** | **11** | **12** | **13** | **14** | **15** | **16** | **17** | **18** | **19** | **20** | **21** | **22** | **23** | **24** | **25** | **26** | **27** |  |
| **Corrective** | | | | | | | | | | | | | | | | | | | | | | | | | | | | | |
| Chen et al (2015)[16] | 2  RCT | Y | Y | Y | Y | Y | Y | Y | N | Y | N | UTD | UTD | Y | N | N | Y | UTD | Y | N | Y | UTD | UTD | UTD | UTD | Y | Y | Y | 57% |
| Kanatli et al (2016)[12] | 2  RCT | Y | Y | N | Y | N | Y | Y | N | Y | Y | UTD | UTD | UTD | N | UTD | Y | N | Y | UTD | Y | UTD | UTD | N | N | Y | Y | N | 43% |
| Wenger et al (1989)[37] | 2  RCT | Y | Y | Y | Y | P | Y | Y | N | N | N | Y | UTD | Y | N | Y | Y | N | Y | UTD | UTD | Y | Y | Y | N | N | N | Y | 57% |
| **Functional Stability** | | | | | | | | | | | | | | | | | | | | | | | | | | | | | |
| Abd Elkader et al (2013)[14] | 3  Before-after Study | Y | Y | Y | N | P | Y | Y | N |  | Y | UTD | UTD | Y | N | Y | Y |  | Y |  | Y | Y | UTD | N | N | Y |  | N | 58% |
| Aboutorabi et al (2014)[11] | 3  Before-after Study | Y | Y | Y | N | Y | Y | Y | N |  | Y | UTD | UTD | UTD | N | UTD | Y |  | Y |  | Y | UTD | UTD | UTD | N | Y |  | Y | 54% |
| Basta et al (1977)[43] | 3  Before-after Study | Y | Y | N | Y | N | N | N | N |  | N | UTD | UTD | Y | N | UTD | Y |  | N |  | Y | UTD | N | N | N | N |  | N | 25% |
| Jagadamma et al (2009)[39] | 3  Before-after Study | Y | Y | Y | Y |  | Y | Y | N |  | Y | UTD | UTD | UTD | N | N | Y |  | Y |  | Y |  |  | N | N |  |  | N | 53% |
| Knittel and Staheli (1976)[38] | 3  Before-after Study | Y | Y | N | N |  | Y | Y | N |  | N | UD | UD | UD | N | UTD | Y |  | Y |  | Y |  |  | N | N |  |  | N | 36% |
| Wesdock & Edge (2003)[40] | 3  Cross Over Study | Y | Y | Y | Y |  | Y | Y | N | Y | N | UTD | UTD | UTD | N | N | Y | Y | Y | UTD | Y |  |  | N | N |  | Y | N | 52% |
| **Functional Instability** | | | | | | | | | | | | | | | | | | | | | | | | | | | | | |
| Ramstrand et al (2008)[41] | 3  Before-after Study | Y | Y | Y | N |  | Y | Y | N | Y | N | UTD | UTD | UTD | N | N | Y | Y | Y | UTD | Y |  |  | N | N |  | Y | N | 48% |
| **Functional Lift** | | | | | | | | | | | | | | | | | | | | | | | | | | | | | |
| Eek et al (2017)[10] | 3  Before-after Study | Y | Y | Y | Y | Y | Y | Y | N |  | Y | Y | UTD | Y | N | N | Y |  | Y |  | Y | Y | UTD | N | N | Y |  | N | 67% |
| Zabjek et al (2001)[42] | 3  Before-after Study | Y | Y | N | N |  | Y | Y | N |  | N | UTD | UTD | Y | N | N | Y |  | Y |  | Y |  |  | N | N |  |  | Y | 47% |

Yes (Y) Score = 1 except Item 5 score = 2 No (N) Score = 0 Partially (P) Score = 1 Unable to determine (UTD) Score = 0 , RCT Randomised Control Trial
