# Supplementary material for: Effectiveness of therapeutic footwear for children: A systematic review
Source: J Foot Ankle Res. 2020 May 13;13:23. doi: 10.1186/s13047-020-00390-3 (PMC7222438; doi:10.1186/s13047-020-00390-3)
Supplement: Supplementary file 5 — Additional file 5. Level of evidence and quality assessment of survey study. [file 13047_2020_390_MOESM5_ESM.docx]

Additional File 5: Level of evidence and quality assessment of survey study.

| **Author Year** | **Level of Evidence**  **Study Design**  **OCEBM** | **Quality Assessment Burns and Kho Assessment Tool for Surveys** | | | | | | | | | | | | | | | | | | | | | |
| --- | --- | --- | --- | --- | --- | --- | --- | --- | --- | --- | --- | --- | --- | --- | --- | --- | --- | --- | --- | --- | --- | --- | --- |
|  |  | 1)  Question | 2)  Population | | 3)  Develop Questionnaire | | | 4)  Testing Questionnaire | | 5)  Administration | | 6)  Response | | | | 7)  Results Reporting | | | | | | | |
|  |  | 1 a | 2a | 2b | 3a | 3b | 3c | 4a | 4b | 5a | 5b | 6a | 6b | 6c | 6d | 7a | 7b | 7c | 7d | 7e | 7f | 7g | 7h |
| **Functional Stability** | | | | | | | | | | | | | | | | | | | | | | | |
| Bakker (1997)[45] | 4  Survey | Y | Y | Y | N | Y | N | N | N | Y | N | Y | Y | UTD | Y | Y | N | N | Y | Y | Y | Y | Y |

Y =Yes, N =No
